# The Fragility of Statistically Significant Results in Randomized Clinical Trials for COVID-19

**DOI:** 10.1001/jamanetworkopen.2022.2973

**Published:** 2022-03-18

**Authors:** Takahiro Itaya, Yotsuha Isobe, Sayoko Suzuki, Kanako Koike, Masakazu Nishigaki, Yosuke Yamamoto

**Affiliations:** 1 Department of Healthcare Epidemiology, Graduate School of Medicine and Public Health, Kyoto University, Kyoto, Japan; 2 Department of Human Health Sciences, Graduate School of Medicine, Kyoto University, Kyoto, Japan; 3 Department of Medical Genetics, International University of Health and Welfare Graduate School, Tokyo, Japan

## Abstract

**Importance:**

Interpreting results from randomized clinical trials (RCTs) for COVID-19, which have been published rapidly and in vast numbers, is challenging during a pandemic.

**Objective:**

To evaluate the robustness of statistically significant findings from RCTs for COVID-19 using the fragility index.

**Design, Setting, and Participants:**

This cross-sectional study included COVID-19 trial articles that randomly assigned patients 1:1 into 2 parallel groups and reported at least 1 binary outcome as significant in the abstract. A systematic search was conducted using PubMed to identify RCTs on COVID-19 published until August 7, 2021.

**Exposures:**

Trial characteristics, such as type of intervention (treatment drug, vaccine, or others), number of outcome events, and sample size.

**Main Outcomes and Measures:**

Fragility index.

**Results:**

Of the 47 RCTs for COVID-19 included, 36 (77%) were studies of the effects of treatment drugs, 5 (11%) were studies of vaccines, and 6 (13%) were of other interventions. A total of 138 235 participants were included in these trials. The median (IQR) fragility index of the included trials was 4 (1-11). The medians (IQRs) of the fragility indexes of RCTs of treatment drugs, vaccines, and other interventions were 2.5 (1-6), 119 (61-139), and 4.5 (1-18), respectively. The fragility index among more than half of the studies was less than 1% of each sample size, although the fragility index as a proportion of events needing to change would be much higher.

**Conclusions and Relevance:**

This cross-sectional study found a relatively small number of events (a median of 4) would be required to change the results of COVID-19 RCTs from statistically significant to not significant. These findings suggest that health care professionals and policy makers should not rely heavily on individual results of RCTs for COVID-19.

## Key Points

**Question:**

In randomized clinical trials (RCTs) of COVID-19 that report statistically significant results, what is the fragility index, ie, the minimum number of participants who would need to have had a different outcome for the RCT to lose statistical significance?

**Findings:**

In this cross-sectional study of 47 RCTs with a total of 138 235 participants that had statistically significant results, the median fragility index was 4. That is, a median of 4 events was required to change the analysis findings from statistically significant to not significant.

**Meaning:**

In this study, many RCTs for COVID-19 had a low fragility index, challenging confidence in the robustness of the results.

## Teaser

This cross-sectional study evaluates the robustness of statistically significant findings from randomized clinical trials for COVID-19 using the fragility index.

## Introduction

Since December 2019, the number of people with COVID-19 has surged worldwide.^1^ Information about this newly discovered infectious disease has been widely reported in both traditional and social media, resulting in global awareness of a previously unknown respiratory infection and increased public perception of risk. This emergency situation has pressured researchers to conduct randomized clinical trials (RCTs) immediately, at various study scales and of varied quality.^2^ Regardless of the scale and quality of RCTs, the results of each received attention from the general public and health care researchers, via different media, and people alternated between optimism and despair based on the individual findings of these trials.^3^

In particular, there is risk that the results depend on the number of outcome events, as designing a trial for an expected number of outcome events is unrealistic in an emergent situation. *P* values are likely to change if the number of events is small.^4^ Furthermore, *P* values can be affected by methodological limitations, such as loss to follow-up or inadequate blinding. However, there is still a strong reliance on *P* values for quick clinical decisions, despite several statements critiquing the superficial interpretation of *P* values.^5,6^

**Figure 1.**
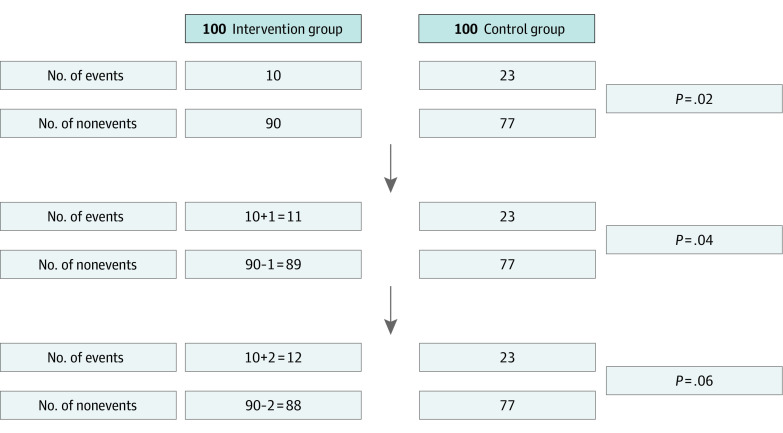
Example of the Fragility Index Calculation for a Randomized Clinical Trial In this example, the original *P* value from the Fisher exact test was .02, and the fragility index was 2. This means that the statistically significant result would not have been significant if 2 cases had changed from nonevents to events in the intervention group.

The fragility index is helpful in interpreting the robustness of results obtained from clinical trials.^7^ It outlines the minimum number of participants in a positive trial who would need to have had a different outcome for the results of the trial to lose statistical significance. A lower number on the fragility index indicates that the statistical significance of the trial depends on fewer events. For example, a score of 2 on this measure means that if 2 participants in the intervention group had different event outcomes, the RCT would not have a statistically significant result when using the conventional *P* value cutoff of less than .05 (Figure 1). Specifically, *P* values from studies with low fragility indexes should be carefully interpreted because they can change easily depending on the number of events. Thus, the fragility index can be an intuitive indicator for the careful interpretation of clinical trial findings conducted under emergency status. The aim of this study was to evaluate the robustness of statistically significant findings from RCTs for COVID-19 using the fragility index.

## Methods

### Study Design and Data Source

For this cross-sectional study, we systematically searched PubMed to identify articles reporting RCTs on COVID-19 until August 7, 2021, using the following search strategy: (*COVID-19* OR *COVID-19* [Medical Subject Heading (MeSH) Terms] OR *COVID-19 Vaccines* OR *COVID-19 Vaccines* [MeSH Terms] OR *COVID-19 serotherapy* OR *COVID-19 serotherapy* [Supplementary Concept] OR *COVID-19 Nucleic Acid Testing* OR *covid-19 nucleic acid testing* [MeSH Terms] OR *COVID-19 Serological Testing* OR *covid-19 serological testing* [MeSH Terms] OR *COVID-19 Testing* OR *covid-19 testing* [MeSH Terms] OR *SARS-CoV-2* OR *sars-cov-2* [MeSH Terms] OR *Severe Acute Respiratory Syndrome Coronavirus 2* OR *NCOV* OR *2019 NCOV* OR *coronavirus* [MeSH Terms] OR *coronavirus* OR *COV*) AND (*randomized controlled trial* [Publication Type] OR (*randomized* [Title/Abstract] AND *controlled* [Title/Abstract] AND *trial* [Title/Abstract])) AND (*2019/11/01* [PDAT]: *3000/12/31* [PDAT]).

Per the Common Rule, this study did not require ethical approval because we analyzed only published results and did not include patients. We followed the Strengthening the Reporting of Observational Studies in Epidemiology (STROBE) reporting guidelines for cross-sectional studies.

### Study Selection

After removing duplicate records from the initial search results, 2 pairs of reviewers (T.I. and K.K.; Y.I. and S.S.) screened the titles and abstracts of all identified articles in accordance with the following prespecified eligibility criteria. The inclusion criteria were RCTs that (1) were superiority trials, (2) randomly assigned patients 1:1 into 2 parallel groups, (3) reported at least 1 dichotomous or time-to-event outcome as statistically significant in the abstract, and (4) tested an intervention for COVID-19. Exclusion criteria were RCTs that were (1) not original articles, (2) preprint articles, (3) phase 1 or 2 trials, (4) noninferiority trials, (5) cluster or crossover RCTs, and (6) non-English articles.

### Data Extraction

The 4 reviewers independently extracted data from each trial in duplicate using a prespecified data collection form. Discrepancies were discussed in pairs; if not resolved, they were addressed by a third reviewer from the review team. We extracted the following data: type of intervention (treatment drug, vaccine, or others); outcome definitions (primary or secondary, time-to-event or not, composite or not); analytical strategy (adjusted confounders or not, intention to treat or not); allocation concealment (adequate or no/unclear); the number of participants lost to follow-up; the reported *P* value; the number of outcome events; the sample size; funding (nonprofit, profit, both, no funding, or not reported).

### Outcome

The primary outcome of this study was the fragility index. We calculated the fragility indexes in each RCT based on a previous report.^7^ Using 2 × 2 contingency tables, the fragility index was calculated by the iterative addition of an event to the experimental or control group with a smaller number of events and concomitant subtraction of a nonevent from that same group. We continued this calculation until statistical significance (defined as *P* < .05) was lost, while maintaining the total number of events and nonevents. *P* values were recalculated using a 2-sided Fisher exact test. In terms of time-to-event outcome, based on previous studies,^7^ we calculated the fragility index by the number of events and nonevents during the observation period, without considering censoring.

### Statistical Analysis

To summarize study characteristics, continuous variables are presented as medians with IQRs, and categorical variables are presented as counts with percentages. We plotted the fragility index as a histogram and described the fragility index by subgroups based on trial characteristics. All statistical analyses were performed using Stata version 16.1 (StataCorp).

## Results

### Selection Flow

**Figure 2.**
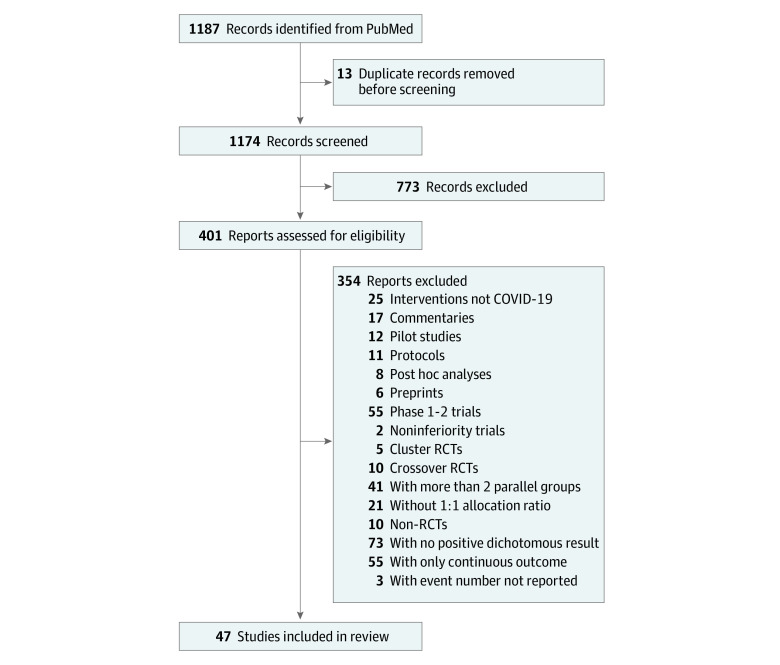
Study Selection Flow RCT indicates randomized clinical trial.

We identified 1187 articles. After excluding duplicate articles and applying the exclusion criteria, 401 articles were deemed eligible for the full-text review. These articles were checked according to the eligibility criteria, and 47 articles, with 138 235 participants, were included in the study.^8,9,10,11,12,13,14,15,16,17,18,19,20,21,22,23,24,25,26,27,28,29,30,31,32,33,34,35,36,37,38,39,40,41,42,43,44,45,46,47,48,49,50,51,52,53,54^ At the full-text review stage, 73 articles were studies with binary outcomes but were excluded because they did not have statistically significant results. The detailed study selection flow is presented in Figure 2.

### Study Characteristics

Table 1 summarizes the characteristics of the included studies. Of the 47 RCTs, 36 (77%) were studies of the effects of treatment drugs, 5 (11%) were vaccines, and 6 (13%) were other topics. The median (IQR) sample size was 111 (72-392) participants, with a median (IQR) of 44 (18-112) outcome events. Approximately half the trials were conducted based on nonprofit funding.

**Table 1.** Characteristics of Included Studies

Characteristic	Studies, No. (%) (N = 47)
Intervention	
Treatment drugs	36 (77)
Vaccines	5 (11)
Others	6 (13)
Outcome	
Primary	23 (49)
Secondary	24 (51)
Time-to-event	6 (13)
Composite	7 (15)
Total sample size, median (IQR)	111 (72-392)
Loss to follow-up, median (IQR)	3 (0-37)
Outcome events, median (IQR), No.	44 (18-112)
Reported *P* value	
<.05-.01	22 (47)
<.01-.001	9 (19)
<.001	11 (23)
Unclear (eg, reported only 95% CI)	5 (11)
Intention-to-treat analysis	25 (53)
Adjusted analysis	8 (17)
Allocation concealment	40 (85)
Funding	
Nonprofit	24 (51)
Profit	5 (11)
Both	6 (13)
No funding	8 (17)
Not reported	4 (9)

### The Fragility Index in COVID-19 Trials

**Figure 3.**
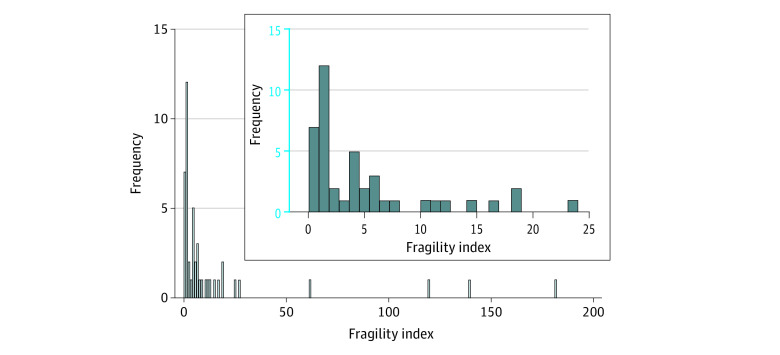
Distribution of the Fragility Index for All Studies

The median (IQR) fragility index for the 47 trials was 4 (1-11): a median of 4 events was required to change the analysis findings from statistically significant to not significant. Figure 3 shows the distribution of the fragility index for the included studies. We describe the fragility index by subgroups of trial characteristics in Table 2. The median (IQR) fragility indexes of RCTs in treatment drugs was 2.5 (1-6); in others it was 4.5 (1-18). In contrast, the median (IQR) fragility index of vaccine trials was 119 (61-139). In addition, among 26 trials (55%), the fragility index was 1% or less of the total sample size.

**Table 2.** Fragility Index by Subgroups Based on Trial Characteristics

Characteristic	No.	Fragility index, median (IQR)
All trials	47	4 (1-11)
Type of intervention		
Treatment drugs	36	2.5 (1-6)
Vaccines	5	119 (61-139)
Others	6	4.5 (1-18)
Outcome		
Primary	23	5 (1-12)
Not primary	24	1.5 (1-6)
Time-to-event	6	4.5 (4-14)
Not time-to-event	41	3 (1-10)
Composite	7	4 (1-11)
Not composite	40	4 (1-11)
Analysis		
Adjusted	8	9 (4.5-129)
Not adjusted	39	2 (1-8)
Intention to treat	25	4 (1-8)
Not intention to treat	22	1 (1-14)
Allocation concealment		
Adequate	40	3.5 (1-7.5)
Unclear	7	14 (1-61)
Loss to follow-up		
≤1%	18	4 (1-7)
>1%-5%	8	1 (0.5-3)
>5%-10%	9	6 (3-11)
>10%	12	3.5 (1-19)
*P* value		
<.05-.01	22	1 (0-1)
<.01-.001	9	4 (4-6)
<.001	11	12 (6-24)
Unclear	5	61 (4-119)
Outcome events, No.^a^		
6-18	12	1.5 (1-4)
19-44	12	1 (0-7)
45-112	12	5 (1-10)
113-839	11	12 (5-119)
Sample size, No.^a^		
34-72	12	2.5 (0.5-4.5)
73-111	12	1 (1-8)
112-392	12	4 (1-9.5)
393-39 058	11	12 (4-119)
Funding		
Nonprofit	24	3 (1-6)
Profit	5	18 (1-61)
Both	6	5.5 (1-12)
No funding	8	3 (0.5-12.5)
Not reported	4	5.5 (2-16)

^a^ The number of events and sample size were divided by IQR into 4 groups.

## Discussion

Our study found that the fragility index was 4 or less in 50% of binary outcomes from RCTs on COVID-19 reported in medical journals published until the beginning of August 2021. This result means that for half the COVID-19 trials, reversing the outcome status of 4 patients in the intervention group would change the result from statistically significant to not significant. In terms of types of interventions, most COVID-19 vaccine trials had a large fragility index, whereas most RCTs studying treatment drugs and other interventions had a very small fragility index. In addition, the fragility index among most of the studies was less than 1% of each sample size.

Our findings were consistent with those reported in various clinical fields surveyed before the pandemic, such as spine surgery,^55,56^ anesthesia and critical care,^57,58,59^ sports medicine and arthroscopic surgery,^60^ and nephrology.^61^ These previous studies reported a median fragility index of 2 to 5, which is similar to our results. In addition, consistent with that reported in previous studies, the fragility index appeared to be associated with the sample size and *P* values. In this study, the sample size of clinical trials examining vaccines was very large, and the fragility index was large in many of these studies. These RCTs of vaccines not only had large sample sizes, but also a high number of events. This result was consistent with those of previous studies that focused on clinical trials in 5 high-impact medical journals, such as *JAMA *and the *New England Journal of Medicine*,^7^ and in heart failure.^62^ These RCTs also had both large sample sizes and large numbers of outcome events.

We need to carefully interpret the results of COVID-19 trials with a small fragility index. A small fragility index means that the results may be less robust in terms of statistical significance; in other words, a change in the outcome occurrence for a small number of participants in an intervention group can easily change the study result. However, a small fragility index does not imply that the study is not trustworthy. Small RCTs with low fragility indexes may still prove useful if the aggregated or the individual patient data they provide can be combined on evidence synthesis platforms, such as the COVID-NMA project.^63^

### Strengths and Limitations

Our study had several strengths. We used a systematic and rigid approach to identify all RCTs related to COVID-19. We systematically identified the articles using a predefined search strategy for all articles in PubMed, which is the most commonly used medical literature database. In addition, we included all eligible COVID-19 trials, regardless of publication period; this makes our findings relatively comprehensive for COVID-19 research and reflects the overall state of the evidence currently available.

This study also has limitations. First, the concept of the fragility index can only be applied to trials performing 1:1 randomization and reporting statistically significant findings for binary outcomes.^7^ Although many clinically relevant end points have binary outcomes, many articles in this study were excluded because they had more than 2 parallel arms (n = 41), no positive dichotomous outcome (n = 73), and only continuous variables (n = 55). Second, we included only articles written in English. This restriction may have led to selection bias, but as the leading studies on COVID-19 are often published in international journals that are PubMed-listed in English, it is unlikely to have caused major problems. Third, the current study did not assess the study quality and the study protocol of individual RCTs in detail and only focused on the fragility index. We only considered a few major aspects of study quality, such as intention-to-treat analysis and allocation concealment. A study with a large fragility index does not necessarily indicate a good study. A larger sample size is likely to result in a larger fragility index, but ethical considerations require that RCTs recruit the minimum number of participants necessary based on the findings of previous studies. The fragility index is only a metric to ascertain the robustness of clinical trials and should not be used alone to judge the merits of a study. Furthermore, there is no clear cutoff point for the fragility index.^64^ Although we have to pay attention to these limitations, the fragility index is an intuitive aid for interpreting RCT results because the simple metric is easy to interpret and may help allay complex concerns regarding smaller trials with fewer events that are difficult to understand intuitively.

## Conclusions

In this study, we found that the statistically significant findings of many COVID-19 trials depended on few events. Therefore, health care professionals and policy makers should not rely heavily on individual results of RCTs on COVID-19. The fragility of RCT results should be considered before applying them to clinical settings. Nevertheless, small RCTs with low fragility indexes may still provide robust and useful findings using evidence synthesis platforms.
